# Red blood cell distribution width and iron deficiency anemia among pregnant Sudanese women

**DOI:** 10.1186/1746-1596-7-168

**Published:** 2012-12-03

**Authors:** Esam G Abdelrahman, Gasim I Gasim, Imad R Musa, Leana M Elbashir, Ishag Adam

**Affiliations:** 1 Khartoum Teaching Hospital, Khartoum, Sudan; 2 Faculty of Medicine, University of Khartoum, P.O. Box 102, Khartoum, Sudan; 3 Faculty of Medicine, Qassim, University, Qassim, Qassim, Kingdom of Saudi Arabia; 4 Buraidah Central Hospital, Buraidah, Kingdom of Saudi Arabia

**Keywords:** Anemia, Pregnancy, Red cell distribution width, Sudan

## Abstract

**Background:**

Iron deficiency anemia (IDA) is a major health problem during pregnancy and it has adverse effects on the mother and the newborn. Red cell distribution width (RDW), which is a quantitative measure for red cell size variation (anisocytosis), is a predictor of IDA. Little is known regarding RDW and IDA during pregnancy.

**Methods:**

A cross sectional study was conducted at the antenatal clinic of Khartoum Hospital, Sudan, to determine the performance of RDW in the diagnosis of IDA using serum ferritin as a gold standard.

**Results:**

Among 194 pregnant women with a gestational period of 21.4 ± 6.5 weeks, 57 (29.4%) had IDA according to serum ferritin levels (<15 μg/l) and 61 (31.4%) had IDA according to RDW (>14.5). The sensitivity, specificity, positive predictive value, and negative predictive value of RDW where serum ferritin was the gold standard were 43.8% (95% CI: 31.4–57.0%), 73.7% (95% CI: 65.8–80.5%), 41.0% (95% CI: 29.2–53.6%), and 76.0% (95% CI: 68.1–82.6%), respectively.

**Conclusions:**

In this study, we found that RDW has a poor performance in diagnosing IDA among pregnant women compared with serum ferritin as the gold standard.

**Virtual slides:**

The virtual slides for this article can be found here: http://www.diagnosticpathology.diagnomx.eu/vs/1721072967826303

## Background

It has been estimated that the highest proportions of individuals affected by anemia are in Africa, e.g. in neighboring Ethiopia, anemia is a major problem for both pregnant (62.7%) and non-pregnant (52.3%) women of child-bearing age [1]. However, the prevalence of anemia varies significantly both within and between countries, which indicates a need for local data to help improve preventive programs. Anemia during pregnancy is associated with increased maternal morbidity and mortality, and contributes to 20% of the maternal mortality in Africa [2-5]. Anemia is one of the most common nutritional deficiency disorders in the world [6]. The World Health Organization (WHO) defines iron deficiency anemia (IDA) as anemia accompanied by depleted iron stores and signs of a compromised supply of iron to the tissues [7,8].

Anemia during pregnancy is a large health problem in Sudan, where pregnant women in different regions of Sudan are more susceptible to anemia, irrespective of their age or parity [9,10]. Furthermore, anemia is associated with poor maternal and perinatal outcomes, such as maternal and perinatal mortality [10-12].

**Figure 1:**
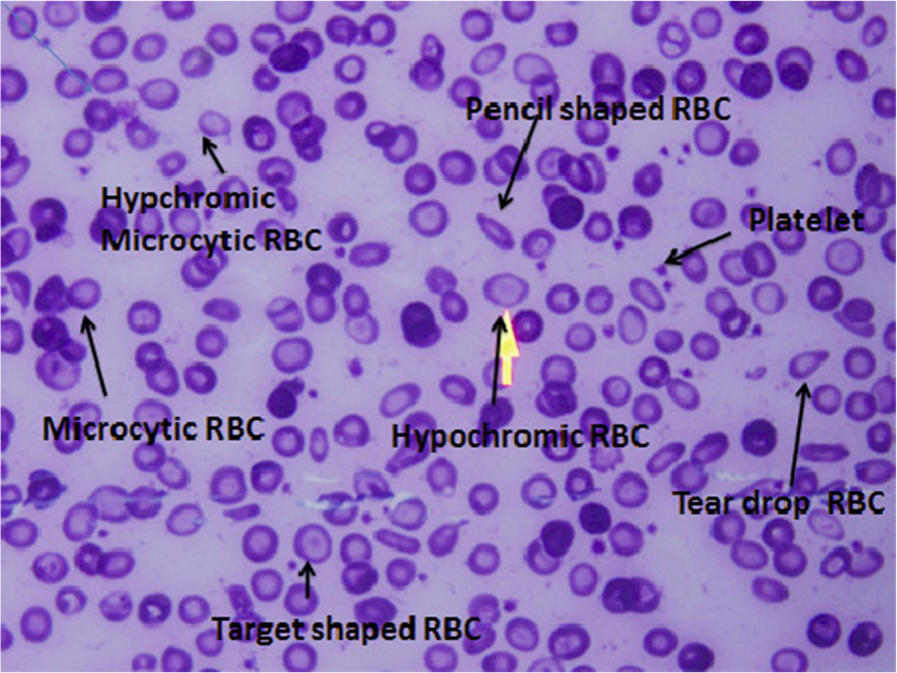
Peripheral blood film with changes attributed to iron deficiency anemia.

Because of physiological changes that occur during pregnancy, some of the hematological parameters, such as mean corpuscular volume (MCV), mean corpuscular hemoglobin (MCH), and mean corpuscular hemoglobin concentration (MCHC), are not sensitive indicators for diagnosing anemia/IDA because they are reduced only when anemia is severe or well established [13]. A peripheral blood film examination provides less information but it requires an expert’s opinion [14,15], and anisocytosis is not sensitive in reflecting IDA because it is less prominent in pregnancy (Figure 1). During normal physiology in pregnancy, serum iron, serum ferritin, and its percentage saturation falls, and total iron binding capacity increases [13-15]. Although serum ferritin is the gold standard for diagnosis of IDA, it is a complicated process, expensive, and being an acute phase reactant, may give misinterpretation because of false elevation [14]. Red cell distribution width (RDW) is a relatively new, routine parameter, which is evaluated in a fully automated hematology analyzer, and is part of the complete blood count (CBC). RDW can reflect early changes in RBCs, which are accompanied by IDA. Therefore, the CBC can be used as a simple and relatively cheap test to detect IDA through the e RDW [16]. RDW expresses small variations and changes in different populations of red cell size [17]. There are few published data on the performance of RDW and IDA during pregnancy [16]. Therefore, the current study was conducted to investigate the performance of RDW in diagnosing IDA in pregnancy, to thus provide information on anemia during pregnancy and its diagnostic tools in Sudan [18].

## Methods

A cross-sectional descriptive study was carried out at the antenatal clinic of Khartoum Hospital, Sudan during the period of January to March, 2012 to investigate the performance of RDW in diagnosing IDA where serum ferritin was the gold standard. A sample size of 190 subjects was calculated based on a 2-sided hypothesis tests using Epiinfo with 80% power and a confidence interval (CI) of 95%. After signing an informed consent, pregnant women attending the antenatal clinic for the first time with singletons were approached to participate in the study.

Those subjects having any of the following: diabetes mellitus, hypertension, taking iron supplements or a history of blood transfusion within the last 3 months were excluded. The obstetric and medical history was determined using pretested questionnaires. A total of 5 ml of blood was collected through an aseptic venipuncture from the antecubital vein. Two milliliters of blood was taken in an ethylene diamine tetra acetic acid and immediately analyzed for a complete hemogram, including hemoglobin, white blood count (WBC), and RDW, using an automated hematology analyzer [19]. Details of the hemoanalyzer are provided in our previous study [18]. In summary, the Sysmex KX21N [19] is an automated blood cell counter intended for in vitro diagnostic use in clinical laboratories. It is a compact, fully automated hematology analyzer with simultaneous analysis of 18 parameters in whole blood mode and capillary blood mode. This analyzer counts blood cells as routine in a few minutes. The test was performed as stated in the manufacturer’s manual [19].

The remaining 3 mls of blood were delivered into a dry clean plain container, centrifuged after clotting and kept at –20 °C until analysis for the serum iron profile. Ferritin levels were measured by immunofluorescent assay using Immulite kits (Siemens, Los Angeles, CA, USA).

IDA was defined as hemoglobin less than 11 g/dl and serum ferritin level (<15 μg/l). Those women with severe anemia (hemoglobin <7 g/dl) and with leukocytosis (WBC >17.1 × 109/L were excluded. [20] RDW >14.5% is considered as abnormal and diagnostic for IDA [17]. The serum ferritin level (<15 μg/l) was the gold standard for the diagnosis of IDA in this study.

### Statistical analysis

The sensitivity, specificity, positive predictive value, and negative predictive value were calculated. The sensitivity of the RDW was calculated as true positives/(true positive + false negatives), the specificity was calculated as true negatives/(true negatives + false positives), the positive predictive value was calculated as true positives/(true positives + false positives), and the negative predictive value was calculated as true negatives/(true negatives + false negatives) [21].

### Ethics

The current study received ethical approval from the Department of Obstetrics in Khartoum Hospital, Sudan.

## Results

During the study period, 212 pregnant women were initially enrolled and 194 fulfilled the inclusion criteria and their data are presented in this report. The characteristics of these women at admission to the study are shown in Table 1. Hemoglobin values ranged from 7.8–15.1 with a mean of 11.7 g/dl. Out of these 194 women, 57 (29.4%) and 61 (31.4%) had IDA according to serum ferritin and RDW cut-off levels for IDA.

**Table 1:** Basic characteristics of the pregnant women included in the study at Khartoum Teaching Hospital, Sudan

**Variables**	**Mean (SD)**
Age, years	25.6 (6.7)
Parity	1.79 (1.8)
Gestational age, weeks	21.4 (6.5)
Weight, kg	73.68 (16.6)
Hemoglobin, gm/dl	11.73 (1.2)
Height/cm	159.5 (6.8)

Among the 57 women that had IDA using serum ferritin level as a diagnostic tool, 25 (43.8%) of them had IDA as indicated by RDW. Therefore, the sensitivity of RDW was 43.8% (95% CI: 31.4–57.0%). Among the 137 women who had no IDA using serum ferritin levels as a diagnostic tool, 36 had IDA by using RDW. The specificity of RDW was 73.7% (95% CI: 65.8–80.5%).

Among those women who had IDA by RDW (61), 25 (41.0%) had IDA according to serum ferritin levels, while 36 did not have IDA. The positive predictive value of RDW was 41.0% (95% CI: 29.2–53.6%). Among those women who did not have IDA by RDW (133), 101 did not have IDA according to low serum ferritin levels, while 32 women had IDA. The negative predictive value of RDW was 76.0% (95% CI: 68.1–82.6%, Tables 2 and 3).

**Table 2:** Results of RDW compared with serum ferritin levels for diagnosis of IDA among pregnant women at Khartoum Hospital, Sudan

**IDA according to the serum ferritin result**	**IDA according to RDW results**			
		**IDA**	**without IDA**	**Total**
	IDA	25	32	57
	without IDA	36	101	137
Total		61	133	194

**Table 3:** Performance of RDW in the diagnosis of IDA among pregnant women at Khartoum Hospital

**Validity test Percentage**	**(95% CI)**
Sensitivity	43.8 (31.4–57.0)
Specificity	73.7 (65.8–80.5)
Positive predictive value	41.0 (29.2–53.6)
Negative predictive value	76.0 (68.1–82.6)

## Discussion

The main findings of the current study were the low sensitivity (43.8%) and moderate specificity (73.7%) of the RDW in the diagnosis of IDA among pregnant women in Sudan. Previously, hemoglobin was the most commonly used hematological parameter and screening test for IDA [22]. However, hemoglobin has its limitation in detecting IDA because sufficient time must elapse for iron to have an effect [23], and hemoglobin may take up to 2 months to show low levels [24]. A recent study showed that other hematological parameters, which can be estimated via a hemoanalyzer, such as MCV, MCH and MCHC, have poor performance in detecting IDA during pregnancy [16]. It is possible that these red cell indices (MCV, MCH and MCHC) are mean values, which cannot express the small variation of red cell size that occurs in early iron deficiency [25]. Changes in the peripheral blood film (e.g., erythrocyte hypochromia and microcytosis) are less prominent during pregnancy than during the non-pregnant condition, even in moderate iron deficiency [26]. Therefore, there is a need for a screening test that is cheap and has a high reliability and accuracy for identifying iron deficiency.

A recent observation among non-pregnant women of child-bearing age showed that RDW (≥16.1%) had a sensitivity of 59.3% and specificity of 71% [27]. Another study among pregnant women in the first half of pregnancy (<20 weeks gestation) showed that an RDW ≥15 had a sensitivity and specificity of 46.8% and 95.7%, respectively [28]. A high sensitivity (82.3%) and specificity (97.4%) for RDW was reported recently among pregnant women [16]. Different rates of RDW have been reported. For example, Aulakh et al. [29] found that the sensitivity of RDW was 81.0% and the specificity was 53.4%, and vanZeben et al. [30] found that the sensitivity of RDW was 94% and the specificity was 59%.

Some points need to be taken into consideration when comparing different studies. First, some reports used a ferritin level of 10 μg/l as a cut-off point for diagnosis of iron deficiency [31-33]. Second, there was a difference in the gestational age between our study and previous studies [16,28].

It should be noted that because of the high prevalence of IDA in pregnancy, it is customary in many settings to consider/treat empirically pregnant anemic patients with iron supplements. Therefore, most of the studies, including the current study, were focused and designed to have maximum specificity for diagnosing IDA rather than achieving the most sensitivity.

This study has some limitations. Hemoglobin electrophoresis, serum vitamin B12, and folate tests were not performed. Such tests are important for excluding hemoglobinopathies, early macrocytosis due to folic acid, or vitamin B12 deficiency where RDW may increase. Anemia of chronic disorders also could not be excluded by an appropriate investigation in cases where serum ferritin levels might have been misinterpreted. The other parameters of iron status or profile such as serum iron level, total iron binding capacity and transferrin saturation were not investigated. Screening for submicroscopic malaria (as it is one of the commonest causes of anaemia) was not performed.

## Conclusion

In this study; RDW has a poorer performance than serum ferritin in diagnosing IDW among pregnant women.

## Competing interests

The authors declare that they have no competing interest.

## Authors’ contributions

EG, GIG and IA designed the study. ERM and LME conducted the laboratory work. GIG and IA analyzed and interpreted the data. All authors drafted and critically revised the manuscript and approved the final version of the paper.
